# Artificial Intelligence in Type 1 Diabetes Management: A Scoping Review of Randomised Controlled Trials

**DOI:** 10.1111/dom.70671

**Published:** 2026-03-19

**Authors:** Yucen Wu, Jun Pang, Shaoyong Xu, Lalantha Leelarathna, Aaron M. Lett

**Affiliations:** ^1^ Department of Metabolism Digestion and Reproduction, Faculty of Medicine, Imperial College London London UK; ^2^ Centre for Clinical Evidence‐Based and Translational Medicine, Xiangyang Central Hospital, Affiliated Hospital of Hubei University of Arts and Science Xiangyang Hubei China; ^3^ Drug Discovery Biology, Monash Institute of Pharmaceutical Sciences, Monash University Melbourne Australia; ^4^ Department of Endocrinology Xiangyang Central Hospital, Xiangyang, Affiliated Hospital of Hubei University of Arts and Science Xiangyang Hubei China; ^5^ Imperial College Healthcare NHS Trust London UK

**Keywords:** artificial intelligence, diabetes management, randomised controlled trials (RCTs), safety, type 1 diabetes

## Abstract

**Background:**

Artificial intelligence is emerging in healthcare systems. In type 1 diabetes, AI‐enabled tools are increasingly used to support nutrition assessment and insulin decision‐making, yet their clinical utility and safety remain unclear.

**Methods:**

The study aims to identify and map the evidence on the clinical utility of AI‐based diabetes management tools in people with type 1 diabetes. We conducted a scoping review following PRISMA‐ScR guidelines, searching PubMed, CINAHL and Web of Science up to January 2026 for eligible randomised controlled trials.

**Results:**

Our findings indicate that the evidence base is small and concentrated in high‐income settings, with most trials assessing clinical utility using CGM outcomes and showing mixed improvements across interventions. No serious safety events were reported, but small sample sizes, short follow‐up and inconsistent safety reporting limit confidence.

**Conclusions:**

Future research should prioritise larger, longer‐term real‐world evaluations that use standardised safety endpoints and patient‐centred outcomes, including in low‐ and middle‐income countries to support equitable implementation.

## 1 Introduction

Type 1 diabetes mellitus (T1DM) is an autoimmune disease characterised by immune‐mediated destruction of pancreatic β cells, resulting in lifelong absolute insulin deficiency and the need for continual day‐to‐day self‐management to achieve and maintain safe glycaemic control [1, 2, 3]. In 2025, an estimated 9.5 million people are living with Type 1 diabetes (T1D) globally, with prevalence expected to reach 14.7 million by 2040 [4]. If glycaemic management is inadequate, T1DM can lead to acute and long‐term complications, including macrovascular events such as heart attack and stroke, and progressive damage to organs as shown in microvascular diseases, including diabetic nephropathy, retinopathy or neuropathy [5].

Artificial intelligence (AI) has rapidly advanced in recent decades [6]. AI is emerging across healthcare with improved facilities and greater computing power, particularly for pattern recognition and neural networking support [7]. However, in current diabetes care, algorithms are primarily model‐based to support day‐to‐day glycaemic management, for instance, in continuous glucose monitoring [8]. The use of AI although emerging, is still within its infancy, particularly within the context of its clinical utility [8].

Even with continuous glucose monitoring and hybrid closed‐loop insulin delivery, mealtime management remains a major source of residual glycaemic dysregulation and self‐management burden. Postprandial glucose excursions are a key driver of overall glycaemic exposure, and CGM‐based analyses suggest that post‐meal periods can contribute substantially to HbA1c in T1DM [9]. Hybrid closed‐loop systems still underperform in the postprandial phase because they rely on manual meal‐related inputs (e.g., bolus timing and carbohydrate estimates) and must contend with delayed subcutaneous insulin kinetics and complex meal responses [10]. In routine care, accurately estimating meal carbohydrate content remains challenging and can translate into clinically meaningful insulin dosing error [11]. Recent trials evaluating simplified meal announcements versus exact carbohydrate counting highlight both the persistence of this burden and the clinical relevance of reducing reliance on precise manual estimation, strengthening the rationale for AI‐enabled meal assessment and decision support as an important target for evaluation [12].

However, the clinical utility of AI in T1DM management, particularly its effects on patient outcomes and safety, remains unclear as comparatively few studies have evaluated these endpoints. In this scoping review, we aim to identify and map the evidence on the clinical utility of AI‐based diabetes management tools in people with type one diabetes, with a focus on patient outcomes and safety. In particular, we will place emphasis on evidence from randomised controlled trials to identify potential directions for future research.

## 2 Method

### 2.1 Study Design

A scoping review was chosen because we anticipated a limited and heterogeneous evidence base on the use of AI in diabetes management for people with T1DM. The review was conducted in accordance with the JBI Manual for Evidence Synthesis and reported following the PRISMA Extension for Scoping Reviews (PRISMA‐ScR) (Supporting Information 1) [13, 14]. We performed comprehensive searches in PubMed, CINAHL and Web of Science from database inception to 1 January 2026, supplemented by targeted grey literature searching to enhance coverage and minimise the risk of missing relevant evidence. The full search strategy can be found in Supporting Information 2.

### 2.2 Inclusion/Exclusion Criteria

The PCC framework (population, concept, context) was used to define study eligibility (Table 1). Population: we included people living with type 1 diabetes of any age, and excluded non‐human studies, studies without type 1 diabetes and mixed diabetes populations where type 1 diabetes outcomes could not be separated. Concept: we included AI‐enabled digital tools used for type 1 diabetes glycaemic management, where AI‐enabled was operationalised as tools that generate patient‐specific estimates, predictions, recommendations, or automated therapy adjustments using computational approaches that go beyond fixed, static rules. Specifically: (i) data‐driven pattern recognition, (ii) adaptive/learning‐based recommendation and/or (iii) predictive/optimisation‐based decision support or automated control. We included interventions reporting endpoints relevant to glycaemic management in type 1 diabetes. We excluded tools that relied solely on manual/user entered input. We also excluded algorithm development, validation, or accuracy‐only studies without a randomised clinical evaluation. Context: we included randomised trials (parallel‐group or crossover) and pilot randomised controlled trials, and excluded all other study designs, including observational studies, non‐randomised trials and reviews.

**TABLE 1:** PCC (population, concept, context) criteria for inclusion and exclusion of studies.

Criteria	Inclusion criteria	Exclusion criteria
Population	People living with type 1 diabetes (T1D) of any age.	Non‐human studies, and studies without T1D or where T1D outcomes cannot be separated from mixed diabetes populations.
Concept	AI‐enabled digital tools for type 1 diabetes glycaemic management, defined operationally as tools that generate patient‐specific estimates/predictions/recommendations or automated therapy adjustments using machine learning/pattern recognition, adaptive learning, and/or predictive/optimisation‐based decision support or automated control based on CGM/pump/meal data.	Tools relied solely on manual/user entered input and/or algorithm development, validation, or accuracy‐only studies without a randomised clinical evaluation
Context	Randomised trials (including parallel‐group or crossover and pilot RCTs).	All other study designs (e.g., observational studies, non‐randomised trials, reviews).

### 2.3 Study Screening

**FIGURE 1:**
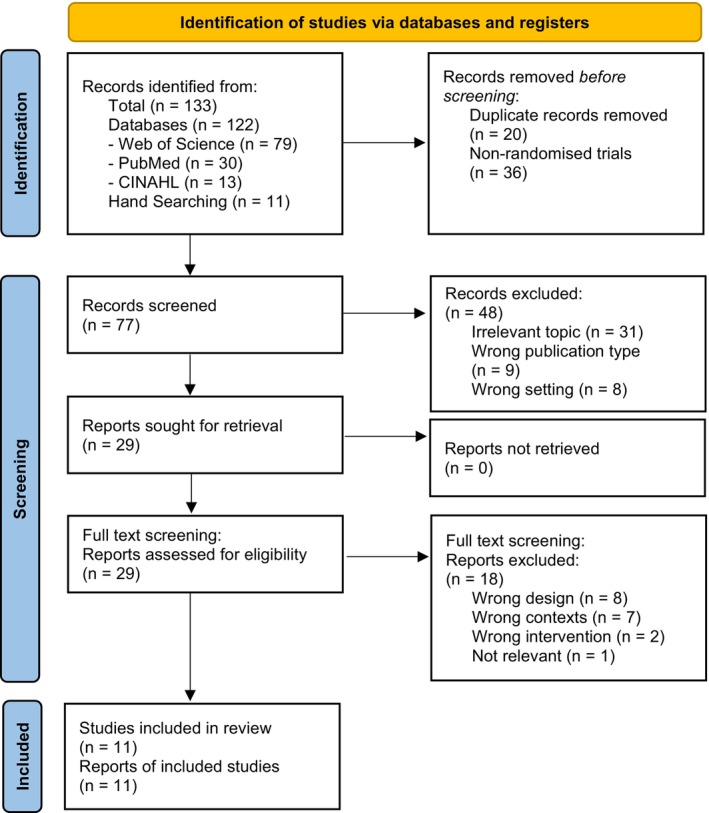
PRISMA Flowchart. *Source:* M.J. Page, et al. *BMJ* **372** (2021): n71, https://doi.org/10.1136/bmj.n71 . This work is licensed under CC BY 4.0. To view a copy of this licence, visit https://creativecommons.org/licenses/by/4.0/.

Two reviewers (Y.W. and J.P.) conducted independent literature screening using the PCC framework, and both reached agreement on the papers included for analysis. The Covidence systematic review software (Veritas Health Innovation; Melbourne, Australia) was used for the removal of duplicates and in exporting relevant reference lists. Studies were initially screened based upon their title and abstract [15]. This was then followed by a full‐text review in potentially eligible cases. In cases of disagreement, a third independent reviewer (AL) was available to resolve differences and decide about inclusion or exclusion. The reasons for exclusion were recorded in accordance with the guidelines outlined in the JBI Manual for Evidence Synthesis [13]. A PRISMA flowchart of studies used is shown in Figure 1. A dedicated data extraction form was established with Microsoft Excel (Microsoft Corporation; Redmond, WA, USA) with all entries made by YW and JP independently verified by AL.

### 2.4 Risk of Bias Assessment

Risk of bias was assessed independently by two reviewers (JP and SX) using the Cochrane Risk of Bias 2 (RoB 2) tool for randomised trials, applying the RoB 2 crossover extension where applicable [16]. A third reviewer (AL) oversaw the process and resolved disagreements. RoB 2 evaluates five domains: (1) bias arising from the randomisation process, (2) bias due to deviations from intended interventions, (3) bias due to missing outcome data, (4) bias in measurement of the outcome and (5) bias in selection of the reported result; for crossover trials, an additional domain addressing bias arising from period and carryover effects was assessed. Each domain was judged as low risk, some concerns, or high risk of bias, and these judgements were combined to derive an overall risk‐of‐bias judgement for each study outcome. As this review was a scoping review and was not designed to undertake quantitative effect synthesis or meta‐analysis, RoB 2 appraisals were conducted as a supplementary methodological assessment to support interpretation of the randomised evidence rather than to generate pooled effect estimates or exclude studies.

### 2.5 Data Extraction

The included studies were first classified into crossover and parallel‐group RCTs. We extracted key study characteristics, including author, year, country/region, study design, population and sample size, to contextualise the evidence base (Table 2). We then extracted outcome‐relevant data from each study, including AI/algorithmic tool, patient‐reported outcomes, safety outcomes and key findings, to inform the subsequent discussion (Table 3).

**TABLE 2:** Characteristics of studies included in the scoping review (*n* = 11) in order according to date of publication (oldest to newest).

Study and population characteristics				
[Ref.]; Author and years	Country/region	Study design	Population	Sample size
Crossover				
[17]; Palisaitis et al., 2020	Canada	Three‐way RCT	Adolescents aged 12–18 years	13 admitted, 11 included for analysis
[18]; Avari et al., 2021	United Kingdom	Open‐label, multicentre RCT	Adults (≥ 18 years) with T1D ≥ 1 year, on MDI (basal–bolus) or CSII/pump ≥ 6 months, HbA1c 48–86 mmol/mol, carb‐counting competent	58 enrolled; 54 completed run‐in (ITT); 50 randomised (PEPPER first *n* = 24/Control first *n* = 26)
[19]; Jacobs et al., 2023	USA	RCT	Adults with type 1 diabetes, aged 21–50; T1D ≥ 1 year; on insulin pump; able to perform aerobic exercise; HbA1c ≤ 10%	27 enrolled, 25 participated; 24 completed exMPC arm, 22 completed exAPD arm; 24 included in analysis
[20]; Mosquera‐Lopez et al., 2023	USA	RCT	Adults with T1D (≥ 1 year), age 18–65, insulin pump users (stable settings), HbA1c ≤ 10.5%	15 randomised; 13 analysed
[21]; Unsworth et al., 2023	United Kingdom	RCT	Adults with T1D ≥ 3 years, on intensified MDI ≥ 6 months, HbA1c 53–75 mmol/mol (7.0%–9.0%)	37 randomised; 33 analysed
[22]; Kovatchev et al., 2025	USA	RCT	Adults with type 1 diabetes, 18–70 years; insulin pump users ≥ 6 months	77 recruited; 72 completed (Group A *n* = 38, Group B *n* = 34)
[23]; Pryor et al., 2025	USA	RCT	Adults 18–60 with T1D ≥ 1 year, using insulin pump ≥ 3 months (open‐loop or HCL) and Dexcom G6/G7 CGM	*n* = 6 randomised and completed (8 consented; 1 screen fail; 1 withdrew)
Parallel‐groups				
[24]; Alfonsi et al., 2020	Canada	Pilot RCT	Youth with type 1 diabetes, aged 10–17 years	Randomised *n* = 46 (23/group); baseline characteristics reported for *n* = 22 iSpy vs. *n* = 22 control; follow‐up outcomes reported *n* = 21 iSpy vs. *n* = 22 control
[25]; Nimri et al., 2020	Multi‐country	2‐arm RCT	Youth with T1D (diagnosis ≥ 1 year), aged 10–21, on insulin pump therapy	112 planned; primary paired analysis limited to 56 pairs.
[26]; Bisio et al., 2022	USA	2‐arm RCT	Adolescents/adults with T1D, age > 15 years, on MDI + CGM	*n* = 80
[27]; Piazza et al., 2025	Switzerland	Open‐label RCT	Adults (≥ 18 years) with T1D (duration ≥ 6 months), HbA1c ≤ 12%, using commercial hybrid AID in Switzerland for ≥ 3 months	44 randomised (22 SNAQ vs. 22 control); primary endpoint analysed *n* = 43 (one control had no AID export data)

Abbreviations: CGM = continuous glucose monitor; MDI = metered‐dose inhaler.

**TABLE 3:** Characteristics of studies included in the scoping review (*n* = 11) in order according to date of publication (oldest to newest).

Study and population characteristics		Study outcomes		
[Ref.]; Author and years	AI/algorithmic tool	Patient‐reported outcomes	Safety	Key outcomes
Crossover				
[17]; Palisaitis et al., 2020	Automated Meal Assessment (ML/CV); Predictive AID Control (MPC‐based)	Not reported	1 hypoglycaemic event (occurring in the AP arm)	**CGM‐derived glycaemic outcomes:** TIR (3.9–10.0 mmol/L), 0–4 h post‐lunch (%): AP + MDA 40.9% ± 27.9% vs. CSII 20.5% ± 27.5% (*p* = 0.03). AP alone was 25.0% ± 19.7% and did not differ vs. CSII (*p* = 0.61) or vs. AP + MDA (*p* = 0.07). TAR (> 10.0 mmol/L), 0–4 h post‐lunch (%): AP + MDA 58.0% ± 26.6% vs. CSII 79.6% ± 27.5% (*p* = 0.02) and vs. AP alone 74.2% ± 20.6% (*p* = 0.047). TBR (< 3.9 mmol/L), 0–4 h post‐lunch (%): 0% [IQR 0–0] in all three arms (CSII, AP, AP + MDA). CGM glucose: Glucose at 4 h post‐lunch, where AP + MDA was 8.5 [6.9–10.5] mmol/L vs. CSII 14.4 [12.9–17.2] mmol/L (*p* = 0.01)
[18]; Avari et al., 2021	Adaptive Bolus Recommenders (CBR)	**Quality of life** PAID: Control 15.6 (9.7–24.4) vs. PEPPER 17.5 (10.0–28.8); *p* = 0.44. QoL global: Control 1.7 (1.3–2.1) vs. PEPPER 1.7 (1.4–2.0); *p* = 0.80 (subscales all NS). **Treatment burden** DTSQs global satisfaction: Control 32 (28–33) vs. PEPPER 31 (28–34); *p* = 0.83. **Fear of hypoglycaemia** DTSQs perceived hypoglycaemia frequency: Control 2 (1–2) vs. PEPPER 2 (2–4); *p* = 0.03 (↑perceived hypo with PEPPER).	2 SAEs occurred in the control condition (one severe nocturnal hypoglycaemia; one mild DKA precipitated by denatured insulin + UTI)	**CGM‐derived glycaemic outcomes:** TIR: 3.9–10.0 mmol/L (70–180 mg/dL); Run‐in 55.0 (46.4–65.6)%; Control 58.4 (49.6–64.3)%; PEPPER 62.5 (52.1–67.8)%; *p* = 0.27. TBR: < 3.9 mmol/L (< 70 mg/dL) Run‐in 2.7 (1.6–5.5)%, Control 2.3 (1.1–6.4)%, PEPPER 2.2 (1.5–3.3)%, *p* = 0.64; < 3.0 mmol/L (< 54 mg/dL) Run‐in 1.0 (0.4–1.9)%, Control 0.4 (0.1–2.0)%, PEPPER 0.4 (0.2–1.1)%, *p* = 0.84. TAR: > 10.0 mmol/L (> 180 mg/dL) Run‐in 42.8 (30.1–49.3)%, Control 38.6 (30.6–48.0)%, PEPPER 35.2 (29.8–43.9)%, *p* = 0.40; > 15.0 mmol/L (> 270 mg/dL) Run‐in 6.8 (3.0–12.6)%, Control 4.9 (2.7–9.4)%, PEPPER 4.5 (2.2–7.5)%, *p* = 0.50. Mean (CGM): Mean sensor glucose (mmol/L); Run‐in 9.6 (8.6–10.3); Control 9.2 (8.8–10.1); PEPPER 9.0 (8.7–9.7); *p* = 0.56. **Longer‐term glycaemic indicators:** HbA1c (ITT pooled; median [IQR]): mmol/mol—Run‐in 61.0 (57.5–66.1); Control 58.5 (53.0–63.4); PEPPER 58.5 (54.0–61.9); *p* = 0.82; %—Run‐in 7.7 (7.5–8.2); Control 7.5 (7.0–7.9); PEPPER 7.5 (7.1–7.7)
[19]; Jacobs et al., 2023	Predictive AID Control (MPC‐based)	**Treatment burden** exMPC no user prompt/confirmation; exAPD requires user to respond to exercise prompt (METs > 4.0).	Not reported	**CGM‐derived glycaemic outcomes:** *Entire 76 h study* TIR: 3.9–10.0 mmol/L; exAPD 75.5 (10.7)%; exMPC 71.2 (16.1)%; difference −4.3 (16.4); *p* = 0.13. TBR: < 3.9 mmol/L; exAPD 1.30 (2.16)%; exMPC 0.96 (1.21)%; difference −0.33 (1.92); *p* = 0.47. TAR: > 10.0 mmol/L; exAPD 23.2 (10.9)%; exMPC 27.8 (16.0)%; difference 4.6 (16.5); *p* = 0.10. Mean (CGM): mmol/L; exAPD 8.5 (0.9); exMPC 8.8 (1.3); difference 0.32 (1.3); *p* = 0.17.
[20]; Mosquera‐Lopez et al., 2023	Automated Meal Assessment (ML/CV); Predictive AID Control (MPC‐based)	**Treatment burden** Insulin bolus adjusted by participant/investigator 54.5% of true detections; mean absolute change 0.35 U.	Not reported	**Carbohydrate counting/estimation performance** Meal detection sensitivity 83.3%; false discovery rate 16.6%; detection time 25.9 min; meal size estimation via 5 CHO classes; **CGM‐derived glycaemic outcomes:** (Postprandial 0–4 h): TAR > 10.0 mmol/L −10.8% vs. MPC (*p* = 0.04); TIR 3.9–10.0 mmol/L + 9.1% (*p* = 0.09); TBR < 3.9 mmol/L NS (95% CI −0.7 to 2.3; *p* = 0.52); TBR < 3.0 mmol/L NS (*p* = 0.46).
[21]; Unsworth et al., 2023	Adaptive Bolus Recommenders (CBR)	**Quality of life** PAID: baseline 18.8 (10–41.3); change vs. baseline: control −1.25 (−5 to +5) vs. ABC4D +1.3 (−2.5 to 9.4); *p* = 0.62. **Treatment burden** DTSQ total: baseline 28 (22–31); change vs. baseline: control +3.5 (0.5–9.5) vs. ABC4D +2 (−3 to 9); *p* = 0.03. **Fear of hypoglycaemia** (DTSQ perceived): baseline 2 (1–3); change vs. baseline: control 0 (−0.5 to 0.5) vs. ABC4D 0 (0 to 1); *p* = 0.31.	4 AEs, none related to the intervention.	**CGM‐derived glycaemic outcomes:** TIR (3.9–10.0 mmol/L): change +0.1 (−2.6 to +4.0)% (ABC4D) vs. +1.9 (−3.8 to +10.1)% (control); *p* = 0.53. TBR: < 3.9 mmol/L change −0.1 (−0.7 to +0.6)% vs. −0.1 (−1.1 to +0.5)%; *p* = 0.85; < 3.0 mmol/L change 0.0 (−0.2 to +0.1)% vs. −0.1 (−0.3 to 0.0)%; *p* = 0.84; < 2.8 mmol/L change −0.1 (−0.2 to 0.0)% vs. −0.1 (−0.4 to 0.0)%; *p* = 0.93. TAR: > 10 mmol/L change −0.8 (−3.4 to +3.0)% vs. −2.8 (−9.9 to +5.3)%; *p* = 0.49; > 13.9 mmol/L change +0.5 (−1.8 to +3.5)% vs. −0.2 (−8.4 to +3.6)%; *p* = 0.28. Mean glucose (mmol/L): change +0.1 (−0.5 to +0.5) vs. −0.5 (−0.8 to +0.7); *p* = 0.26. **Longer‐term glycaemic indicators:** HbA1c: baseline median (IQR) 61.0 (58.0–67.0) mmol/mol [7.7% (7.5–8.3)]; change (ABC4D vs. control) +2 (−2 to +6) vs. −2 (−5 to +2) mmol/mol, *p* = 0.11 (*n* = 17 with complete HbA1c set). GMI: change (ABC4D vs. control) +1.1 (−2.2 to +2.2) vs. −1.1 (−2.2 to +3.3) mmol/mol, *p* = 0.57 (*n* = 33).
[22]; Kovatchev et al., 2025	AI Dosing Decision Support (DSS)	**Engagement** Logins to ABC system: 0.9 ± 0.4 times/day.	Not reported	**CGM‐derived glycaemic outcomes:** TIR (%), 3.9–10.0 mmol/L: Group A (Escalation; *n* = 38): AID 72.26 (12.65); AID+IF 72.49 (11.77); AID+ABC 76.80 (9.20); Group B (De‐escalation; *n* = 34): AID+ABC 77.91 (10.41); AID+IF 77.93 (9.40); AID 78.03 (10.52) TBR (%), < 3.9 mmol/L: Group A: AID 1.68 (1.74); AID+IF 1.68 (1.50); AID+ABC 1.98 (1.62); Group B: AID+ABC 1.90 (1.27); AID+IF 2.09 (1.57); AID 1.97 (1.74) TAR (%), > 10 mmol/L: Group A: AID 26.84 (13.63); AID+IF 27.57 (12.81); AID+ABC 22.34 (9.52); Group B: AID+ABC 20.81 (9.70); AID+IF 21.01 (9.40); AID 21.00 (10.55) Mean CGM glucose (mmol/L): Group A: AID 8.58 (1.16); AID+IF 8.54 (1.09); AID+ABC 8.16 (0.73); Group B: AID+ABC 8.05 (0.80); AID+IF 8.04 (0.76); AID 8.02 (0.84) **Longer‐term glycaemic indicators:** HbA1c: Baseline (screening): 6.8% ± 0.8% (range 5.3%–9.2%); End of study: 6.6% ± 0.5% (range 5.6%–7.8%)
[23]; Pryor et al., 2025	Neural Controller for Closed‐Loop (Embedded AI)	Not reported	Not reported	**CGM‐derived glycaemic outcomes:** TIR: 3.9–10.0 mmol/L; Usual care 63.9% ± 14.9%; AIDANET 66.4% ± 8.3%; Difference + 2.5 [−8.29, 13.34]; FCL days 67.7% ± 9.4% (vs usual care Difference + 3.8 [−10.12, 17.77]). TBR: < 3.9 mmol/L; Usual care 0.9% ± 1.0%; AIDANET 1.6% ± 1.8%; Difference +0.7 [−1.67, 3.07]; FCL days 1.8% ± 2.0% (Difference + 0.9 [−1.59, 3.45]); < 3.0 mmol/L; Usual care 0.1% ± 0.2%; AIDANET 0.3% ± 0.6%; Difference +0.2 [−0.46, 0.85]; FCL days 0.3% ± 0.6% (Difference +0.2 [−0.48, 0.86]). TAR: > 10.0 mmol/L; Usual care 35.2% ± 15.2%; AIDANET 32.0% ± 8.8%; Difference −3.2 [−13.78, 7.33]; FCL days 30.5% ± 9.8% (Difference −4.8 [−18.31, 8.79]); > 13.9 mmol/L; Usual care 10.3% ± 8.9%; AIDANET 9.7% ± 6.3%; Difference −0.6 [−8.15, 6.86]; FCL days 8.2% ± 8.0% (Difference −2.2 [−12.26, 7.96]). Mean (CGM): mean CGM (mmol/L); Usual care 9.3 ± 1.4; AIDANET 9.0 ± 0.9; Difference −0.4 [−1.5, 0.7]; FCL days 8.8 ± 1.0 (Difference −0.5 [−1.8, 0.8]). **Longer‐term glycaemic indicators:** GMI (%, mean ± SD; last 7 days AIDANET vs. 7 days usual care): Usual care 7.3 ± 0.6; AIDANET 7.2 ± 0.4; Difference −0.2 [−0.63, 0.31]; FCL days 7.1 ± 0.4; Difference −0.2 [−0.78, 0.33].
Parallel‐groups				
[24]; Alfonsi et al., 2020	Automated Meal Assessment (ML/CV)	**Quality of life** No between‐group difference (*p* = 0.64) **Engagement** Usability Acceptability E‐Scale mean 4.6 (SD 0.7); end‐of‐study engagement 43% (9/21) medium/high use.	Not reported	**Longer‐term glycaemic indicators:** HbA1c (%), mean (SD): Baseline iSpy 8.41 (1.84) vs. usual care 8.35 (1.32); *p* = 0.91; 3‐month follow‐up iSpy 8.06 (1.43) (*n* = 21) vs. usual care 8.80 (1.60) (*n* = 22); *p* = 0.03; baseline‐adjusted coefficient −0.603. **Carbohydrate counting/estimation performance:** Absolute error (% of total grams per meal): Baseline iSpy 31.97 (11.36) vs. usual care 32.03 (10.01); *p* = 0.99; Follow‐up iSpy 27.45 (10.90; *n* = 21) vs. usual care 38.00 (14.74; *n* = 22); *p* = 0.008; baseline‐adjusted coefficient −10.479. Counting errors > 10 g (% of items): Baseline iSpy 25.00 (14.06) vs. usual care 27.73 (15.10); *p* = 0.54; Follow‐up iSpy 21.43 (16.82) vs. usual care 32.27 (16.31); *p* = 0.047; baseline‐adjusted coefficient −9.851. Time to complete counting task (seconds): Baseline iSpy 79.95 (23.88) vs. usual care 80.73 (27.82); *p* = 0.92; Follow‐up iSpy 74.86 (31.78) vs. usual care 78.23 (44.97); *p* = 0.79; baseline‐adjusted coefficient −2.741.
[25]; Nimri et al., 2020	AI DSS	Not reported	0 in AI‐DSS vs. 2 severe hypoglycaemia +1 DKA in physician arm.	**CGM‐derived glycaemic outcomes** At 24 weeks (mmol/L): TIR 70%–180%: 50.2 ± 11.1 (AI‐DSS) vs. 51.6 ± 11.3 (physician) (non‐inferior). TBR < 54%: 1.3 ± 1.4 vs. 1.0 ± 0.9 (non‐inferior). TBR < 70%: 3.9 ± 2.7 vs. 3.4 ± 2.2. TAR > 180%: 45.9 ± 11.4 vs. 44.9 ± 12.0; TAR > 240%: 21.4 ± 9.6 vs. 20.4 ± 10.5. **Longer‐term glycaemic indicators** HbA1c baseline: 8.4% ± 0.8% both arms. ΔHbA1c baseline → 24w: AI‐DSS −0.32% (*p* = 0.008); physician −0.19% (*p* = 0.22); between‐arm *p* = 0.49.
[26]; Bisio et al., 2022	AI DSS	**Diabetes distress** Intent‐to‐treat: no sig change/difference. Baseline (active vs. nonactive): 2.0 ± 0.8 vs. 2.2 ± 0.9. **Fear of hypoglycaemia** (HFS‐II): intent‐to‐treat: no sig change/difference. Per‐protocol: HFS‐II total ↓ more in active vs. nonactive (*p* = 0.043); worry subscale ↓ more (*p* = 0.016); within active: total *p* = 0.024, worry *p* = 0.017. Baseline HFS‐II total (active vs. nonactive): 41.3 ± 20.4 vs. 36.3 ± 17.6. **Engagement** Technology Expectations vs. Experience (DSS arm): benefits score decreased (expected → experienced) *p* = 0.004; in active users only *p* = 0.030. Baseline expected benefits/burdens (active vs. nonactive): 71.4 ± 12.4 vs. 56.9 ± 21.7; 28.9 ± 15.9 vs. 39.2 ± 16.5.	Not reported	**CGM‐derived glycaemic outcomes** (mmol/L) TIR 3.9%–10.0%: Control 54.9 → 58.2 (*p* = 0.202); DSS 53.3 → 57.7 (*p* = 0.003); between‐group *p* = 0.855. TAR > 10.0%: Control 40.9 → 38.7 (*p* = 0.396); DSS 41.2 → 38.1 (*p* = 0.050); *p* = 0.916. TBR < 3.9%: Control 3.3 → 2.1 (*p* = 0.059); DSS 3.7 → 2.6 (*p* = 0.058); *p* = 0.832. Mean CGM (mmol/L): Control 9.8 → 9.6 (*p* = 0.605); DSS 9.7 → 9.4 (*p* = 0.129). **Longer‐term glycaemic indicators** HbA1c %: Control 7.7 → 7.3 (*p* = 0.009); DSS 7.4 → 7.1 (*p* = 0.001); *p* = 0.844.
[27]; Piazza et al., 2025	Automated Meal Assessment (ML/CV)	**Treatment burden** Baseline CHO estimation perceived burdensome 19/42 (45%); after 3 weeks, self‐reported burden unchanged. **Fear of hypoglycaemia** ‘Heightened hypoglycaemia concerns’ 12/41 (29%). **Engagement** Mean use 1.6 ± 0.8/day (~38.6% of meals); follow‐up optional use 5/39 (12.8%) used at least once; median 0.1 [0.1–0.2]/day among active follow‐up users.	Not reported	**CGM‐derived glycaemic outcomes:** TIR between SNAQ (used 1.6 ± 0.8 per day) and control was 6.6 percentage points in favour of SNAQ (95% CI 2.9 to 10.3, *p* < 0.001). Mean glucose (−0.54 mmol/L, CI −0.9 to −0.2, *p* = 0.004) and TAR (−6.3%, CI −10 to −2.7, *p* = 0.001). **Carbohydrate counting/estimation performance** Carbohydrate estimation quiz (picture‐based) baseline: absolute error 22.8 g [19.0, 26.5] overall (intervention 23.6 [19.0, 26.2] vs. control 22.8 [19.5, 25.7]); relative error 44% [38.0, 54.0] overall. Deviation between SNAQ‐generated CHO estimates and participant‐entered CHO (during active SNAQ use): participants accepted SNAQ's suggestion in 19.2% of cases and entered lower CHO than suggested in 47.2% of instances; the paper also notes a large proportion of entries deviated by > 20 g from the app estimate.

Abbreviations: AID = automated insulin delivery; CBR = case‐based reasoning; CGM = continuous glucose monitoring; DKA = diabetic ketoacidosis; GMI = glucose management indicator; LSTM = long short‐term memory; MDA = meal‐detection algorithm; MPC = model predictive control; PAID = problem areas in diabetes; QoL = quality of life; SAE = severe adverse effect; TAR = time above range; TBR = time below range; TIR = time in range; UTI = urinary tract infection.

## 3 Results

We conducted a scoping review to identify and map published evidence on the clinical utility of AI in the management of people with type 1 diabetes. Figure 1 presents the PRISMA flow diagram of the screening process. In total, 133 records were retrieved from PubMed, CINAHL, Web of Science and grey literature. After removal of 20 duplicates and exclusion of 36 non‐randomised trials, 77 records were screened by title and abstract. Of these, 48 were excluded due to irrelevant topics, incorrect publication type, or inappropriate setting, leaving 29 records for full‐text review. Following full‐text screening, 8 records were excluded for incorrect study design, 7 for wrong contexts, 2 for inappropriate interventions and 1 for lack of relevance. Ultimately, 11 studies were included for data extraction.

A total of 11 studies met the inclusion criteria. All 11 studies were randomised: 7 used a crossover design, and 4 were parallel‐groups randomised controlled trials. The studies included countries that encompassed the UK [18, 21], USA [19, 20, 22, 23, 26], Canada [17, 24] and Switzerland [27], with 1 multi‐country setting [25]. Table 2 shows the characteristics of the included studies. Overall, included studies focused on people with type 1 diabetes across three broad age groups: adolescents only (12–18 years [17]; 10–17 years [24]), youth/young people spanning adolescence into early adulthood (10–21 years [25]; > 15 years [26]) and adults (≥ 18 years or adult ranges such as 18–60/65/70 or 21–50 years [18, 19, 20, 21, 22, 23, 27]), with several adult studies explicitly enrolling working‐age cohorts [19, 20, 22, 23].

Table 3 summarises the 11 included studies and reports study/population characteristics alongside study outcomes across 4 domains: AI/algorithmic tool, patient‐reported outcomes, safety and key outcomes. The included evidence comprised studies in 5 distinct areas: evaluating automated meal assessment (ML/CV) [17, 20, 24, 27], predictive AID control (MPC‐based) [17, 19, 20], adaptive bolus recommenders (case‐based reasoning) [18, 21], AI dosing decision support systems [22, 25, 26] and an embedded neural controller for closed‐loop insulin delivery [23].

### 3.1 Key Outcomes

#### 3.1.1 Carbohydrate Counting/Estimation Performance

Carbohydrate counting/estimation performance was reported in three studies. Alfonsi et al. reported lower follow‐up absolute error and fewer large errors (> 10 g) in the iSpy group than usual care, while time to complete the task did not differ [24]. Piazza et al. reported baseline picture‐based quiz performance (absolute and relative error) and, during active app use, participants accepted the tool's CHO suggestion in 19.2% of entries and entered lower CHO than suggested in 47.2%, with many deviations > 20 g [27]. Mosquera‐Lopez et al. reported algorithm performance for meal handling, including meal detection sensitivity, false discovery rate and detection time, with meal size estimation using five CHO classes [20].

#### 3.1.2 CGM‐Derived Glycaemic Outcomes

CGM‐derived outcomes were commonly reported as TIR/TAR/TBR and mean CGM glucose. Palisaitis et al. reported higher post‐lunch TIR and lower TAR for AP + MDA versus CSII, with TBR 0% across arms [17]. Avari et al. reported no between‐group differences in CGM‐derived outcomes comparing PEPPER with control [18]. Jacobs et al. reported similar whole‐study CGM outcomes between exAPD and exMPC [19]. Mosquera‐Lopez et al. reported a postprandial TAR reduction versus MPC, with TIR change not statistically significant and TBR non‐significant [20]. Unsworth et al. reported no between‐group differences in CGM‐derived outcome changes between ABC4D and control [21]. Nimri et al. reported non‐inferior CGM outcomes for AI‐DSS compared with physician management at 24 weeks [25]. Bisio et al. reported within‐group changes in CGM metrics over time in both DSS and control, with no significant between‐group differences reported for these CGM outcomes [26]. Kovatchev et al. reported CGM outcomes across escalation and de‐escalation sequences involving AID, AID+IF and AID+ABC [22]. Pryor et al. reported comparable CGM outcomes between usual care and AIDANET (including a subset of fully closed‐loop days) [23]. Piazza et al. reported higher TIR and lower mean glucose and TAR in the meal‐support app group compared with control [27].

#### 3.1.3 Longer‐Term Glycaemic Indicators (HbA1c/GMI)

Longer‐term indicators were reported as HbA1c and/or GMI in 6 studies. Alfonsi et al. reported lower HbA1c at 3‐month follow‐up in the iSpy group compared with usual care (*p* = 0.03) [24]. Nimri et al. reported HbA1c reductions in both arms from baseline to 24 weeks, with no between‐arm difference (*p* = 0.49) [25]. Bisio et al. reported HbA1c reductions over time in both control and DSS groups, with between‐group comparisons reported as non‐significant [26]. Kovatchev et al. reported HbA1c at baseline and end of study [22]. Pryor et al. reported GMI for usual care versus AIDANET (including fully closed‐loop days) [23]. Unsworth et al. reported HbA1c and GMI change from baseline, with no statistically significant between‐group differences reported for these indicators [21].

### 3.2 Patient‐Reported Outcomes

#### 3.2.1 Quality of Life

Quality‐of‐life outcomes were reported in two studies. Alfonsi et al. reported no between‐group difference in quality of life (*p* = 0.64) [24]. Unsworth et al. reported no between‐group difference in diabetes‐related quality‐of‐life distress (PAID) change from baseline (*p* = 0.62) [21].

#### 3.2.2 Treatment Burden

Treatment burden was reported in five studies using measures of satisfaction/interaction burden and user workload. Avari et al. reported no between‐group difference in diabetes treatment satisfaction (DTSQs global satisfaction; *p* = 0.83) [18]. Unsworth et al. reported a between‐group difference in treatment satisfaction change (DTSQ total change; *p* = 0.03) [21]. Jacobs et al. reported that exMPC required no user prompt/confirmation, whereas exAPD required a user response to an exercise prompt (METs > 4.0) [19]. Mosquera‐Lopez et al. reported that bolus doses were adjusted by participant/investigator in 54.5% of true detections (mean absolute change 0.35 U) [20]. Piazza et al. reported that 45% (19/42) perceived CHO estimation as burdensome at baseline, with self‐reported burden unchanged after 3 weeks [27].

#### 3.2.3 Fear of Hypoglycaemia

Fear of hypoglycaemia‐related outcomes were reported in 3 studies. Avari et al. reported a between‐group difference in perceived hypoglycaemia frequency (DTSQs perceived hypoglycaemia frequency), with higher perceived frequency in the PEPPER group (*p* = 0.03) [18]. Unsworth et al. reported no between‐group difference in perceived hypoglycaemia frequency change from baseline (*p* = 0.31) [21]. Piazza et al. reported ‘heightened hypoglycaemia concerns’ in 29% (12/41) of participants [27].

#### 3.2.4 Diabetes Distress

Diabetes distress was assessed in 1 study. Alfonsi et al. reported no between‐group difference in diabetes distress outcomes (non‐significant) [24].

#### 3.2.5 Engagement

Engagement was reported in 3 studies. Piazza et al. reported mean app use of 1.6 ± 0.8 times/day (approximately 38.6% of meals), and 12.8% (5/39) used the tool at least once during optional follow‐up [27]. Kovatchev et al. reported 0.9 ± 0.4 logins/day to the ABC system [22]. Alfonsi et al. reported a Usability Acceptability E‐Scale mean of 4.6 (SD 0.7), with 43% (9/21) classified as medium/high engagement at end of study [24]. Bisio et al. reported that the Technology Expectations vs. Experience ‘benefits’ score decreased from expected to experienced (*p* = 0.004; active users only *p* = 0.030) [26].

### 3.3 Safety

Safety reporting was variable across the 11 trials. Six studies reported no safety events/outcomes [19, 20, 22, 23, 24, 27]. Across the remaining studies, reported events were primarily described as hypoglycaemia/hyperglycaemia episodes or adverse events (AEs)/serious adverse events (SAEs) with attribution by trial arm.

SAEs were reported in 2 trials. In PEPPER, 2 SAEs occurred in the control condition (one severe nocturnal hypoglycaemia and one mild DKA requiring hospital admission, precipitated by denatured insulin with urinary tract infection), and both resolved without sequelae [18]. In ADVICE4U, 3 severe diabetes‐related AEs (two severe hypoglycaemia and one DKA) were reported in the physician arm, with none in the AI‐DSS arm [25].

Non‐serious AEs were reported in 2 trials. In ABC4D, there were no SAEs and no episodes of severe hypoglycaemia, but 4 AEs were recorded; none was deemed related to the study intervention [21]. In PEPPER, 2 additional AEs (appendicitis requiring appendicectomy and a fall resulting in fractured fifth metatarsal) were reported, and neither was deemed related to the PEPPER safety system or AI algorithm [18].

In the meal detection algorithm trial, 1 hypoglycaemic event occurred (in the AP arm), with time < 3.9 mmol/L reported as 0% across arms; hyperglycaemia events requiring correction boluses were also reported across arms (CSII 5, AP 4, AP+MDA 1) [17].

### 3.4 Risk of Bias Assessment

**FIGURE 2:**
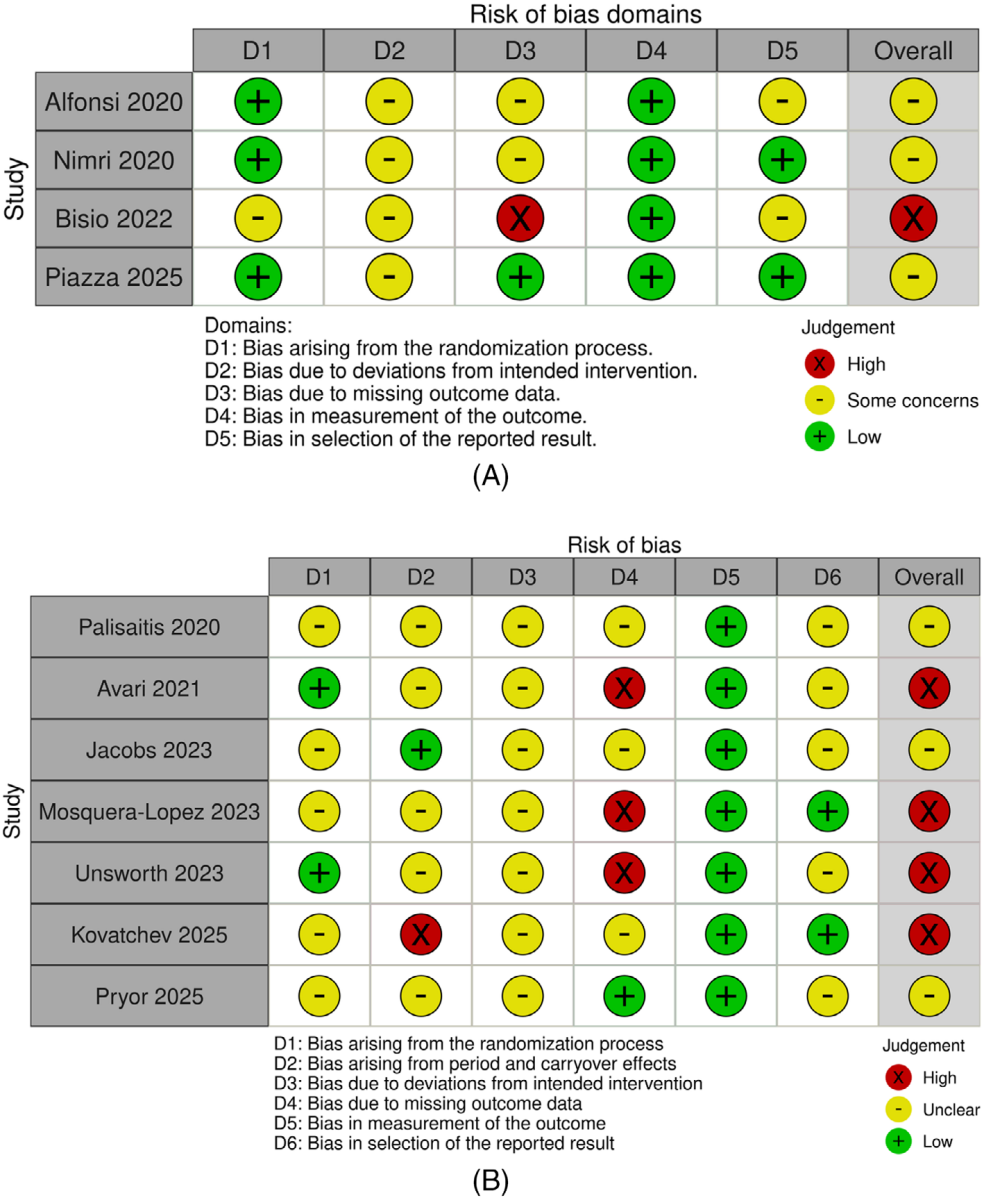
Risk of bias assessment (*n* = 11). General and overall risk of bias for the 11 studies examined using the (A) Cochrane risk‐of‐bias tool (RoB2) and (B) RoB2 with additional considerations for crossover trials.

Figure 2 summarises the RoB 2 assessments for the 11 included studies using design‐appropriate RoB 2 tools (including the crossover extension where applicable) [16]. Six of the 11 studies (55%) were judged to have some concerns of bias, and the remaining 5 (45%) were judged to be at high risk of bias.

## 4 Discussion

This scoping review mapped available literature on the clinical utility and safety of AI‐based diabetes management tools in type 1 diabetes patients, focusing on randomised evaluations of AI‐enabled digital tools involving endpoints relevant to glycaemic management in T1D. The included 11 evidence comprised studies evaluating automated meal assessment (ML/CV) [17, 20, 24, 27], predictive AID control (MPC‐based) [17, 19, 20], adaptive bolus recommenders (case‐based reasoning) [18, 21], AI dosing decision support systems [22, 25, 26] and an embedded neural controller for closed‐loop insulin delivery [23]. Safety reporting, where provided, generally captured serious AEs, severe hypoglycaemia and DKA, but was heterogeneous across trials, limiting cross‐study comparability and reinforcing the need for larger, longer, standardised evaluations that integrate both clinical benefit and safety endpoints in real‐world use [18, 19, 21, 23, 24, 27].

To address the heterogeneity of the AI tools captured by this scoping review, findings should be interpreted by intervention type rather than as a single pooled AI effect, as mechanisms, intended users and risk–benefit profiles differ substantially across applications. The most promising signals currently lie in areas where evidence remains weakest: the clearest short‐term glycaemic improvements are most apparent in mealtime support tools that reduce carbohydrate‐estimation burden and target postprandial control [17, 20, 24, 27], whereas bolus recommenders and dosing decision‐support systems more often show mixed or null effects on primary glycaemic endpoints [18, 21, 22, 25, 26]. Predictive AID control and embedded controllers remain comparatively early‐stage, with limited trial volume and follow‐up, restricting inference beyond proof‐of‐concept [17, 19, 20, 23]. This categorised synthesis strengthens interpretation by showing which AI use‐cases most warrant prioritised, adequately powered pragmatic evaluation, rather than implying similar benefit across all AI‐enabled diabetes management tools.

Given the clinical intuition that contemporary AID remains predominantly hybrid and still depends on user meal input and insulin bolus behaviours [18, 19, 21, 23, 24, 27]. This focus also aligns with wider implementation experience that achieving the benefits of hybrid closed‐loop systems in routine care requires sustained user engagement and training, particularly around meal bolusing and expectation management [28, 29]. In parallel, the reliance on CGM‐derived endpoints (especially TIR) across the included studies reflects the field's convergence on consensus CGM targets as clinically meaningful metrics for technology evaluation [8, 30].

In the context of contemporary type 1 diabetes care, ‘safety’ is judged first and foremost by whether an intervention increases sentinel harms, particularly severe hypoglycaemia (events requiring external assistance) and diabetic ketoacidosis, alongside systematic capture of AEs and serious AEs [8, 31]. Across the 11 randomised trials mapped in this scoping review, sentinel safety events were uncommon and, where they occurred, were reported in control conditions or adjudicated as not related to the AI intervention, with no consistent signal that automated meal assessment or adaptive bolus recommenders increased severe hypoglycaemia or DKA within the trial settings. This is a materially important observation given that a dominant barrier to adoption is not simply efficacy uncertainty, but apprehension that algorithmic recommendations could amplify dosing errors at meals. At the same time, the evidence should be interpreted as bounded reassurance rather than definitive proof of safety: the trials were generally small and short, and safety ascertainment and reporting were heterogeneous (including at least one paediatric study where clinical safety endpoints were not clearly reported), which limits the ability to exclude rare but clinically consequential harms. Framed against this, the current evidence base supports conditional trust in the evaluated AI dosing decision support systems or embedded neural controller for closed‐loop insulin delivery as adjuncts under user and clinical oversight, while underscoring why transparency and non‐reliance principles in clinical decision support are central to safety governance and user confidence [32]. This balanced suggestion is consistent with broader public sentiment: surveys indicate substantial discomfort with healthcare providers relying on AI for diagnosis or treatment recommendations, highlighting that ‘safety’ for AI in diabetes management is as much about demonstrable harm signals and explainability as it is about glycaemic endpoints [33].

Clinical utility in this evidence base should be interpreted through the lens of external validity, because most interventions were evaluated in settings that only partially reflect how AI tools are likely to be used in routine life. Across the 11 randomised trials, participants were generally selected for a relatively technology‐capable context (e.g., carbohydrate‐counting competence, established pump use or established hybrid AID use), and several studies incorporated structured procedures that reduce real‐world noise, including run‐in phases, supervised elements, or short controlled observation windows. Within these conditions, outcome improvement was mixed rather than uniform across tool categories: one computer‐vision nutrition trial in adults using commercial hybrid AID reported a clinically interpretable short‐term improvement in TIR, particularly postprandially, but also documented declining engagement and lack of sustained benefit after discontinuation, which directly qualifies how useful the tool may be in day‐to‐day practice. By contrast, both insulin decision‐support crossover trials did not demonstrate significant glycaemic benefit on their primary endpoints, and the wearable‐integrated algorithm comparison similarly showed no clear between‐algorithm advantage on key CGM endpoints, suggesting that incremental utility may be harder to realise when baseline care is already optimised or when the behavioural component (acting on recommendations) becomes the limiting factor. This behavioural dependence is underscored by the ABC4D trial's lower acceptance of recommended meal doses versus control dosing behaviours, which indicates that real‐world clinical utility is contingent not only on algorithmic performance but also on user trust and adoption. Finally, the paediatric pilot study suggests potential utility on a different axis, namely improved carbohydrate counting accuracy and a modest HbA1c difference, but the pilot nature and endpoint selection limit direct comparison with CGM‐centric adult trials [18, 19, 21, 23, 24, 27]. Taken together, the mapped evidence does not support a conclusion that AI ‘should not’ be pursued in type 1 diabetes management; rather, it indicates that demonstrable utility is presently tool‐ and context‐dependent, and that effectiveness observed under supportive trial conditions may attenuate in real‐world use when engagement, workflow burden and willingness to follow AI advice become decisive determinants of benefit.

A key implication of the mapped evidence is that clinical utility is tightly coupled to human factors because these AI tools typically operate as adjuncts that require active use and discretionary adherence rather than fully autonomous glycaemic control. In the included trials, engagement and behavioural uptake varied in ways that plausibly mediate effectiveness: app use declined over time in the adult computer‐vision nutrition trial and optional follow‐up use was low [27], while the adaptive insulin bolus calculator trial observed lower acceptance of algorithm‐recommended meal doses compared with control dosing behaviours, indicating that users did not consistently operationalise AI outputs even in trial settings [21]. These patterns are consistent with established experience in hybrid closed‐loop therapy, where psychosocial and human factors remain central because the system automates only part of diabetes work and therefore still depends on user understanding, expectations and day‐to‐day interaction [34].

Patient‐related outcomes (PROs) were also inconsistently captured across the 11 studies, and where measured, signals such as higher perceived hypoglycaemia in PEPPER despite broadly comparable glycaemic metrics illustrate that perceived safety and burden can diverge from CGM endpoints and may influence sustained engagement and trust [18]. Across the evidence base, PRO coverage was fragmented and skewed toward select domains (quality of life, diabetes distress, fear of hypoglycaemia, treatment burden, usability/acceptability and crude engagement metrics), leaving important constructs under‐specified, including decision confidence, cognitive load, alert fatigue, perceived autonomy and stigma, equity of use and downstream behaviours. The clinical significance is straightforward: patient‐related outcomes are not ancillary in type 1 diabetes, because perceived burden, safety and confidence determine whether users keep engaging with AI adjuncts long enough for any glycaemic benefit to materialise, and they shape the risk that CGM summaries alone can miss. Future research should therefore treat patient‐centred outcomes as primary or co‐primary endpoints (e.g., diabetes distress, fear of hypoglycaemia, treatment satisfaction, workload, trust calibration and decisional conflict), paired with objective interaction logs and pre‐specified mediation analyses that test whether burden, trust and workflow‐fit explain effects on TIR and safety. Designs should also include longer follow‐up, reporting of differential engagement by age, health literacy and digital exclusion and mixed‐methods evaluation to surface why users accept, ignore, or override AI suggestions [35, 36, 37].

The mapped trial evidence comes exclusively from high‐income countries and predominantly technology‐supported care contexts, which raises a core equity question: whether AI tools will widen disparities if benefits are only demonstrated, and products are only designed and validated in well‐resourced systems. In lower‐resource settings, implementation barriers are likely to include limited access to CGM/pumps and smartphones, data costs/connectivity, language‐ and culture‐tailored food databases, variable health literacy, constrained dietetic and clinical support for onboarding and weaker governance for safety monitoring and accountability [38, 39, 40]. At the same time, the scarcity of high‐quality RCTs limits confidence in both effectiveness and safety, showing an urgent need for larger trials. Future research should therefore prioritise adequately powered evaluations adopting larger multicentre pragmatic RCTs in low‐ and middle‐income countries (LMICs).

Outcome selection and reporting remain fragmented across tool categories. Adult studies largely prioritise CGM‐derived glycaemic metrics, whereas paediatric research has focused more on carbohydrate counting accuracy. This inconsistency limits cross‐study comparability and tends to reduce utility to glycaemia alone. For AI tools in diabetes self‐management, the field needs outcomes that better reflect real‐life impact for people with T1D, such as behavioural change in meal and dosing decisions, treatment burden, quality of life, fear of hypoglycaemia, diabetes distress, sleep quality, usability and trust and sustained engagement over time. Safety reporting also requires greater consistency, particularly in children and adolescents. Trials should prespecify and clearly report core clinical safety endpoints such as severe hypoglycaemia, DKA and AEs, alongside relevant process indicators such as escalation to clinical support when applicable. Future studies would benefit from adopting a core outcome set with minimum requirements for patient‐reported outcomes, engagement and safety, supported by aligned follow‐up windows and transparent reporting to enable more meaningful synthesis across tools and populations.

In summary, this scoping review indicates that the current randomised evidence on AI in diabetes management in type 1 diabetes is early‐stage. It is concentrated on mealtime support functions and evaluated predominantly in high‐income care settings. Across the mapped trials, serious safety events were uncommon, and no consistent signal emerged that AI meal estimation or decision support increased severe hypoglycaemia or DKA within trial conditions. This reassures that AI tools can function as adjuncts under user and clinical oversight. However, clinical utility was mixed and strongly context‐dependent. Benefits were most apparent when tools reduced mealtime burden or improved postprandial control, but effects were smaller when baseline care was already optimised and when real‐world engagement and adherence to AI recommendations became the main limiting factors. Future studies should examine patient‐related outcomes in more depth, as they are central to effective type 1 diabetes management. The evidence base remains constrained by the scarcity of research, small sample sizes, short follow‐up, heterogeneous outcomes and safety reporting. Notably, the evidence to date comes almost entirely from high‐income countries, underscoring the need for adequately powered evaluations in LMICs. Future research should move beyond proof‐of‐concept efficacy to larger, longer‐term pragmatic studies. These evaluations should use standardised glycaemic, safety, behavioural and patient‐centred outcomes. Explicitly address human factors and trust, and prioritise equity by testing deployable AI tools in diverse and lower‐resource settings with feasible governance and safety surveillance.

## Author Contributions

**Yucen Wu:** conceived the study, designed the search strategy, conducted the literature screening and data extraction, contributed to the Risk‐of‐Bias assessment, as well as writing the first manuscript. **Jun Pang:** independently reviewed the literature and contributed to the Risk‐of‐Bias assessment. **Lalantha Leelarathna:** reviewed the manuscript and provided high‐level comments. **Shaoyong Xu:** provided consultation in T1D‐related questions and contributed to the Risk‐of‐Bias assessment. **Aaron M. Lett:** provided overall supervision, generated ideas for the study, provided feedback and comments for the manuscript. All authors had full access to the data, contributed to the development of the conceptual interpretation, and approved the final version of the manuscript for submission.

## Funding

The authors have nothing to report.

## Conflicts of Interest

L.L. reports having received speaker honoraria from Abbott Diabetes Care, Insulet, Medtronic and Sanofi; was on advisory panels for Abbott, Dexcom, Medtronic and Sanofi; and received research support from Abbott Diabetes Care, Novo Nordisk, Insulet and Dexcom. The other authors declare no conflicts of interest.

## Supporting information


**Data S1:** dom70671‐sup‐0001‐Supinfo1.docx.

**Data S2:** dom70671‐sup‐0002‐Supinfo2.docx.

## Data Availability

All data extracted and analysed during this systematic review are available upon reasonable request to the corresponding author. The full list of included studies, data extraction templates and thematic coding frameworks are provided in the Supporting Information. No individual participant data were used or collected, and all data were derived from publicly accessible sources or published literature. Requests for additional materials related to the conceptual model or search strategy can be accommodated by contacting the corresponding author.
