# Supplementary material for: Artificial Intelligence in Type 1 Diabetes Management: A Scoping Review of Randomised Controlled Trials
Source: Diabetes Obes Metab. 2026 Mar 19;28(6):4860–75. doi: 10.1111/dom.70671 (PMC13146131; doi:10.1111/dom.70671)
Supplement: Supplementary file 2 — Data S2: dom70671‐sup‐0002‐Supinfo2.docx. [file DOM-28-4860-s002.docx]

**Supplementary Material 2. Search Strategy**

**PubMed**

(

("Diabetes Mellitus, Type 1"[Mesh] OR "type 1 diabetes"[tiab] OR T1D[tiab])

AND

(meal*[tiab] OR food*[tiab] OR "carbohydrate counting"[tiab] OR carbohydrate*[tiab] OR CHO[tiab]

OR "carbohydrate estimation"[tiab] OR "portion size"[tiab] OR portion*[tiab] OR macronutrient*[tiab]

OR "meal size"[tiab] OR "meal size estimation"[tiab])

AND

(

"Artificial Intelligence"[Mesh] OR "Machine Learning"[Mesh] OR "Deep Learning"[Mesh]

OR "Neural Networks, Computer"[Mesh]

OR "Image Processing, Computer-Assisted"[Mesh] OR "Pattern Recognition, Automated"[Mesh]

OR "Natural Language Processing"[Mesh] OR "Speech Recognition Software"[Mesh]

OR "Chatbots"[Mesh]

OR artificial intelligence[tiab] OR machine learning[tiab] OR deep learning[tiab]

OR neural network*[tiab] OR computer vision[tiab] OR image recognition[tiab]

OR image-based[tiab] OR food recognition[tiab] OR automated meal analys*[tiab]

OR meal detection[tiab] OR automated meal*[tiab]

OR chatbot*[tiab] OR "conversational agent*"[tiab] OR "conversational AI"[tiab]

OR "virtual assistant*"[tiab] OR "dialog system*"[tiab] OR "dialogue system*"[tiab]

OR "large language model*"[tiab] OR LLM*[tiab] OR "language model*"[tiab]

OR "generative AI"[tiab] OR ChatGPT[tiab] OR GPT*[tiab]

)

AND

(

"Monitoring, Ambulatory"[Mesh] OR "Blood Glucose Self-Monitoring"[Mesh]

OR CGM[tiab] OR "continuous glucose monitor*"[tiab] OR "continuous glucose monitoring"[tiab]

OR "flash glucose monitor*"[tiab] OR FGM[tiab] OR "interstitial glucose"[tiab]

OR "time in range"[tiab] OR TIR[tiab] OR "time above range"[tiab] OR TAR[tiab] OR "time below range"[tiab] OR TBR[tiab]

OR GMI[tiab] OR "glucose management indicator"[tiab]

OR "ambulatory glucose profile"[tiab] OR AGP[tiab]

)

AND

(

insulin[tiab] OR "Insulin"[Mesh]

OR bolus[tiab] OR boluses[tiab] OR "meal bolus"[tiab]

OR "insulin bolus"[tiab] OR "insulin dose"[tiab] OR "insulin dosing"[tiab]

OR "bolus calculator*"[tiab] OR "dose calculator*"[tiab]

OR "insulin-to-carbohydrate"[tiab] OR "insulin to carbohydrate"[tiab] OR ICR[tiab]

OR "correction factor"[tiab] OR "insulin sensitivity factor"[tiab] OR ISF[tiab]

OR "preprandial insulin"[tiab] OR "postprandial insulin"[tiab]

OR "closed-loop"[tiab] OR "hybrid closed-loop"[tiab] OR "automated insulin delivery"[tiab] OR AID[tiab]

)

AND

(randomized controlled trial[pt] OR controlled clinical trial[pt]

OR random*[tiab] OR trial[tiab] OR crossover[tiab] OR "cross-over"[tiab] OR "cross over"[tiab] OR randomi*[tiab]

OR pilot[tiab])

)

NOT (animals[mh] NOT humans[mh])

**CINAHL**

(MH "Diabetes Mellitus, Type 1+") OR TI("type 1 diabetes" OR T1D) OR AB("type 1 diabetes" OR T1D)

AND

TI(meal* OR food* OR "carbohydrate counting" OR carbohydrate* OR CHO OR "carbohydrate estimation" OR "portion size" OR portion* OR macronutrient* OR "meal size" OR "meal size estimation")

OR

AB(meal* OR food* OR "carbohydrate counting" OR carbohydrate* OR CHO OR "carbohydrate estimation" OR "portion size" OR portion* OR macronutrient* OR "meal size" OR "meal size estimation")

AND

(MH "Artificial Intelligence+") OR (MH "Machine Learning") OR (MH "Deep Learning") OR (MH "Neural Networks (Computer)")

OR (MH "Image Processing, Computer Assisted") OR (MH "Pattern Recognition")

OR

TI("artificial intelligence" OR "machine learning" OR "deep learning" OR neural network* OR "computer vision" OR "image recognition" OR image-based OR "food recognition" OR "automated meal analysis" OR text OR speech OR video OR "meal detection" OR "automated meal")

OR

AB("artificial intelligence" OR "machine learning" OR "deep learning" OR neural network* OR "computer vision" OR "image recognition" OR image-based OR "food recognition" OR "automated meal analysis" OR text OR speech OR video OR "meal detection" OR "automated meal")

AND

PT("Randomized Controlled Trial" OR "Clinical Trial" OR "Controlled Clinical Trial")

OR

(MH "Randomized Controlled Trials") OR (MH "Clinical Trials") OR (MH "Crossover Design")

OR

TI(random* OR trial OR crossover OR "cross-over" OR "cross over" OR randomi* OR pilot)

OR

AB(random* OR trial OR crossover OR "cross-over" OR "cross over" OR randomi* OR pilot)

AND

S1 AND S2 AND S3 AND S4

**Web of Science**

TS=("type 1 diabetes" OR T1D OR "diabetes mellitus type 1" OR "type I diabetes")

TS=(meal* OR food* OR "carbohydrate counting" OR carbohydrate* OR CHO OR

"carbohydrate estimation" OR "portion size" OR portion* OR macronutrient* OR

"meal size" OR "meal size estimation")

TS=("artificial intelligence" OR "machine learning" OR "deep learning" OR

"neural network*" OR "computer vision" OR "image recognition" OR

"image-based" OR "food recognition" OR "automated meal analysis" OR

"meal detection" OR "automated meal" OR text OR speech OR video)

TS=("randomized controlled trial" OR "controlled clinical trial" OR random* OR trial OR

crossover OR "cross-over" OR "cross over" OR randomi* OR pilot)
